# From misdiagnosis to definitive diagnosis of SAPHO syndrome during a routine health check-up: a case report and literature review

**DOI:** 10.3389/fmed.2026.1823550

**Published:** 2026-04-28

**Authors:** Yishan Liu, Wenwen Zhao, Jianchen Chen, Yan Wang

**Affiliations:** Department of Health Management Centre, The First Affiliated Hospital of Sun Yat-sen University, Guangzhou, China

**Keywords:** autoinflammatory diseases, case report, diagnostic delay, misdiagnosis, rare diseases, SAPHO syndrome

## Abstract

**Background:**

SAPHO syndrome is a rare chronic autoinflammatory disease with heterogeneous clinical manifestations. Because there are no specific diagnostic biomarkers, the condition is often diagnosed late or misdiagnosed.

**Case presentation:**

A 51-year-old man presented with unexplained chest pain and low back pain. He had been evaluated at several hospitals and had received different diagnoses related to bone disease. During a routine health check-up, imaging revealed multiple osteolytic lesions in the sternum, together with sclerosis and hyperostosis involving the sternoclavicular and sternocostal joints. Laboratory testing showed elevated high-sensitivity C-reactive protein and abnormal rheumatological and immunological indices, suggesting an inflammatory disorder. After admission to the rheumatology department, bone scintigraphy demonstrated the typical “bull’s head sign,” providing supportive imaging evidence. Review of the external bone biopsy report, together with physical examination, laboratory testing, and imaging findings, allowed exclusion of infection, malignancy, and other spondyloarthritides. A definitive diagnosis of SAPHO syndrome was established. The patient was treated with methotrexate, non-steroidal anti-inflammatory drugs and tumor necrosis factor inhibitors, with subsequent pain relief, normalization of inflammatory markers, and stable disease during follow-up.

**Conclusion:**

Because of its non-specific and variable presentation, SAPHO syndrome is frequently misdiagnosed as malignancy, tuberculosis, spondylitis, psoriasis or other conditions. Awareness of the combination of bone and skin manifestations may reduce diagnostic delay. In appropriate clinical settings, bone scintigraphy can provide valuable supportive evidence, but diagnosis should rely on integrated clinical, imaging, and exclusionary assessment. Increased awareness and standardized evaluation may help shorten the diagnostic interval, reduce misdiagnosis and mistreatment, and improve prognosis.

## Introduction

SAPHO syndrome is a rare chronic autoinflammatory disease that mainly affects the skin, bones and joints. It is characterized by inflammatory involvement of the osteoarticular system, often accompanied by skin lesions such as palmoplantar pustulosis and severe acne (1, 2). In 1987, Chamot and colleagues reviewed 85 cases and introduced the term “SAPHO syndrome,” referring to synovitis, acne, pustulosis, hyperostosis and osteitis (1). The pathogenesis remains incompletely understood, and proposed mechanisms include infection, genetic susceptibility, immune dysfunction and environmental influences (3–5). Despite its recognizable clinical spectrum, SAPHO syndrome lacks specific biomarkers, and presents with substantial heterogeneity, which makes diagnosis challenging (5, 6). As a result, diagnostic delay is common, with a mean interval of approximately 9 years from symptom onset to diagnosis (7, 8). Delayed recognition exposes patients to persistent pain, repeated investigations, invasive procedures, and ineffective empiric treatments, including unnecessary anti-tuberculosis or antibiotic therapy.

We report the case of a 51-year-old man with SAPHO syndrome who experienced a 5-year diagnostic odyssey. He was misdiagnosed with spinal tuberculosis and pyogenic spondylitis and received 2 years of unnecessary anti-tuberculosis treatment as well as invasive procedures without benefit. During a routine health check-up, chest computed tomography revealed multifocal osteolysis, sclerosis, and hyperostosis involving the sternum, medial ends of both clavicles, and vertebrae, with involvement of the sternoclavicular and sternocostal joints. Elevated inflammatory markers and abnormal rheumatological and immunological findings suggested an inflammatory disorder. Further evaluation in the rheumatology department, together with bone scintigraphy showing the “bull’s head sign,” supported the diagnosis. The patient improved substantially after treatment with methotrexate, non-steroidal anti-inflammatory drugs, and anti-TNF therapy.

This case highlights the value of routine health check-up in detecting rare diseases and illustrates common diagnostic pitfalls in SAPHO syndrome. We also summarize previously reported cases with diagnostic delay to clarify the recurrent misdiagnostic patterns and key features that may facilitate earlier recognition.

## Case description

A 51-year-old man was referred to the rheumatology department after abnormal findings on a routine health check-up at the Health Management Center of the First Affiliated Hospital of Sun Yat-sen University in October 2022 (Table 1). He had experienced intermittent chest pain for 5 years, first appearing in 2017 and described as a pulling sensation. 4 years before presentation, in 2018, he developed pustular skin lesions on both legs and was diagnosed with pustulosis at a local hospital, but treatment was not continued. 3 years before presentation, in 2019, he developed low back pain and right leg pain without morning stiffness and underwent multiple evaluations without a definitive diagnosis. During this period, erythrocyte sedimentation rate (ESR) and C-reactive protein (CRP) remained persistently elevated. Between 2019 and 2021, bone tuberculosis and spinal tuberculosis were suspected successively, and he received anti-tuberculosis treatment for 2 years, from April 2019 to April 2021, without improvement. Pyogenic spondylitis was later suspected, and he received cephalosporin therapy for 3 months from 2021 to 2022, again without significant relief. A bone biopsy performed at an outside institution on July 21, 2021, tissue NGS reportedly showed chronic osteitis and no evidence of tuberculosis, infection, or malignancy. However, only a written summary of the biopsy was available to us. From 2021 to 2022, the patient reported partial symptom relief with irregular use of over-the-counter celecoxib. He had no significant past medical history, no smoking or alcohol abuse history, and no family history of rheumatic immune diseases or hereditary bone disorders.

**TABLE 1:** Clinical features and diagnostic methods of SAPHO syndrome patients.

Time	Symptoms	Diagnosis	Treatment	Examination items
2017	Intermittent chest pain described as a pulling sensation.	/	/	/
2018 (Hospital 1)	Pustular skin lesions appeared on both legs, accompanied by the above symptoms.	Pustulosis	Hormone therapy was prescribed but not taken by the patient.	/
2019-2021 (Hospital 2–4)	Low back pain and right leg pain without morning stiffness, with recurrent pustular lesions.	Bone tuberculosis, spinal tuberculosis.	Anti-tuberculosis therapy (2019.04—2021.4).	1. PET/CT showed multiple abnormal bone metabolic foci involving the bilateral sternoclavicular joints and sternum. 2. Elevated ESR and CRP. 3. Negative rheumatic immune markers.
2021-2022 (Hospital 5)	Same as before	Pyogenic spondylitis	1. A 3-month course of cephalosporins. 2. Irregular use of over-the-counter celecoxib.	1. Bone biopsy: chronic osteitis, with no evidence of tuberculosis, infections or malignancy (2021.7.21). 2. Persistent elevation of ESR and CRP despite anti-infective therapy.
2022.10 (Our hospital, Health Management Centre).	Same as before	Rheumatological disease	Referral to Department of rheumatology and immunology for further treatment.	1.SAA:79.40 mg/L (↑); IgA:4.68 g/L (↑); IgG:17.40 g/L (↑); C3:1.31 g/L (↑); C4:0.40 g/L (↑); ASO (-); Anti-DNase-B; RF (-); IgM (-). 2. hs-CRP: 38.52 mg/L (↑); WBC: 7.47 × 10^9/L (-). 3. Chest CT: Multiple moth-eaten osteolytic lesions with reactive hyperostosis and sclerosis involving the sternum, medial clavicles.
2022.11 (Our hospital, Department of rheumatology and immunology)	Same as before	Autoinflammatory diseases, SAPHO?	Methotrexate 10 mg PO QW; Celebrex 200 mg PO QD.	ANA, Anti-dsDNA, AHA, AnuA, C1q Ab, Anti-SSA, Anti-SSB, Anti-Sm, Anti-Jo-1, Anti-RNP, Anti-Scl-70, ACA were all negative.
2022.12 (Same as above)	Same as before	SAPHO syndrome	Methotrexate 10 mg PO QW; Celebrex 200 mg PO QD; Adalimumab 40 mg SC BIW.	Bone scintigraphy showed increased tracer uptake in the manubrium, sternal body, bilateral first sternocostal joints, and sternoclavicular joints (“bull’s head sign”).
2023.01 (Same as above)	1. Pustular lesions on the extremities showed significant regression, with most exhibiting crust formation. 2. Chest pain and joint pain were significantly relieved.	Same as before	Same as above	CRP: 0.84 mg/L (-), ESR:16 mm/h (-), WBC: 6.23 × 10^9/L (-).
2023.04 (Same as above)	Same as before	Same as before	Same as above	CRP: 2.63 mg/L (-), ESR: 16 mm/h (-), WBC: 6.79 × 10^9/L (-).
2023.07 (Same as above)	Same as before	Same as before	Same as above	CRP: 2.49 mg/L (-), ESR:28 mm/h (-), WBC: 6.76 × 10^9/L (-).
2023.12 (Routine telephone follow-up from Health Management Centre)	Significant improvement in chest and osteoarticular pain, along with near-complete resolution of their skin lesions.	Same as before	Same as above	/

hs-CRP, hs-CRP, high-sensitivity C-reactive protein (normal < 3 mg/L); CRP, C-reactive protein (normal < 3 mg/L); ESR, erythrocyte sedimentation rate (normal < 28 mm/h); IgG, immunoglobulin G (normal: 10.13–15.13 g/L); IgA, immunoglobulin A (normal: 1.45–3.45 g/L); SAA, serum amyloid A (normal < 6.4 mg/L); C3, complement C3 (normal: 0.79–1.79 g/L); C4, complement C4 (normal: 0.17–0.31 g/L); WBC, white blood cell (normal: 4-10 × 10^9/L); ASO, anti-streptolysin O; Anti-DNase B, anti-DNase B; RF, rheumatoid factor; IgM, immunoglobulin M; ANA, antinuclear antibody; anti-dsDNA, anti-double-stranded DNA antibody; AHA, anti-histone antibody; AnuA, anti-nucleosome antibody; C1q Ab, C1q antibody; anti-SSA, anti-Sjögren’s syndrome A antibody; anti-SSB, anti-Sjögren’s syndrome B antibody; anti-Sm, anti-Smith antibody; anti-Jo-1, anti-Jo-1 antibody; anti-RNP, anti-ribonucleoprotein antibody; anti-Scl-70, anti-Scl-70 antibody; ACA, anticentromere antibody; CT, computed tomography.

**FIGURE 1:**
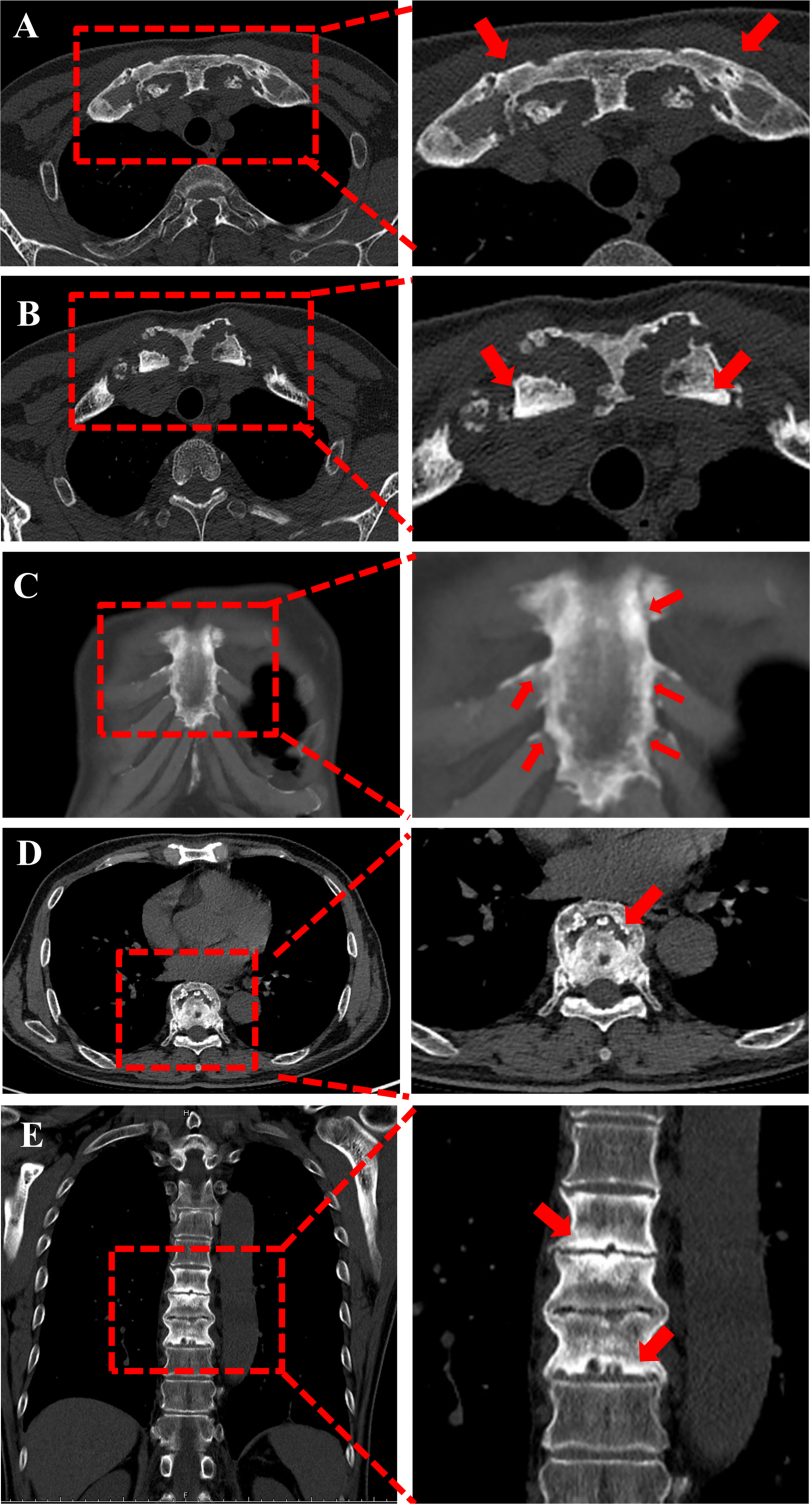
Imaging features of the sternum, clavicles and spine in the present case. CT images were obtained using a bone window setting (window level, C = 500, window width, *W* = 1,500). **(A)** Axial CT shows multiple irregular “worm-eaten” osteolytic lesions in the sternum and bilateral clavicles (arrows). **(B)** Sclerosis and hyperostosis of the sternum and bilateral clavicles are present (arrows). **(C)** Irregular margins of the sternoclavicular joints, sternocostal joints and vertebral endplates are seen. **(D)** Axial images show multiple osteolytic lesions in the thoracic vertebrae (arrows). **(E)** Coronal images show multiple irregular osteolytic lesions in the thoracic and lumbar vertebrae (arrows).

**FIGURE 2:**
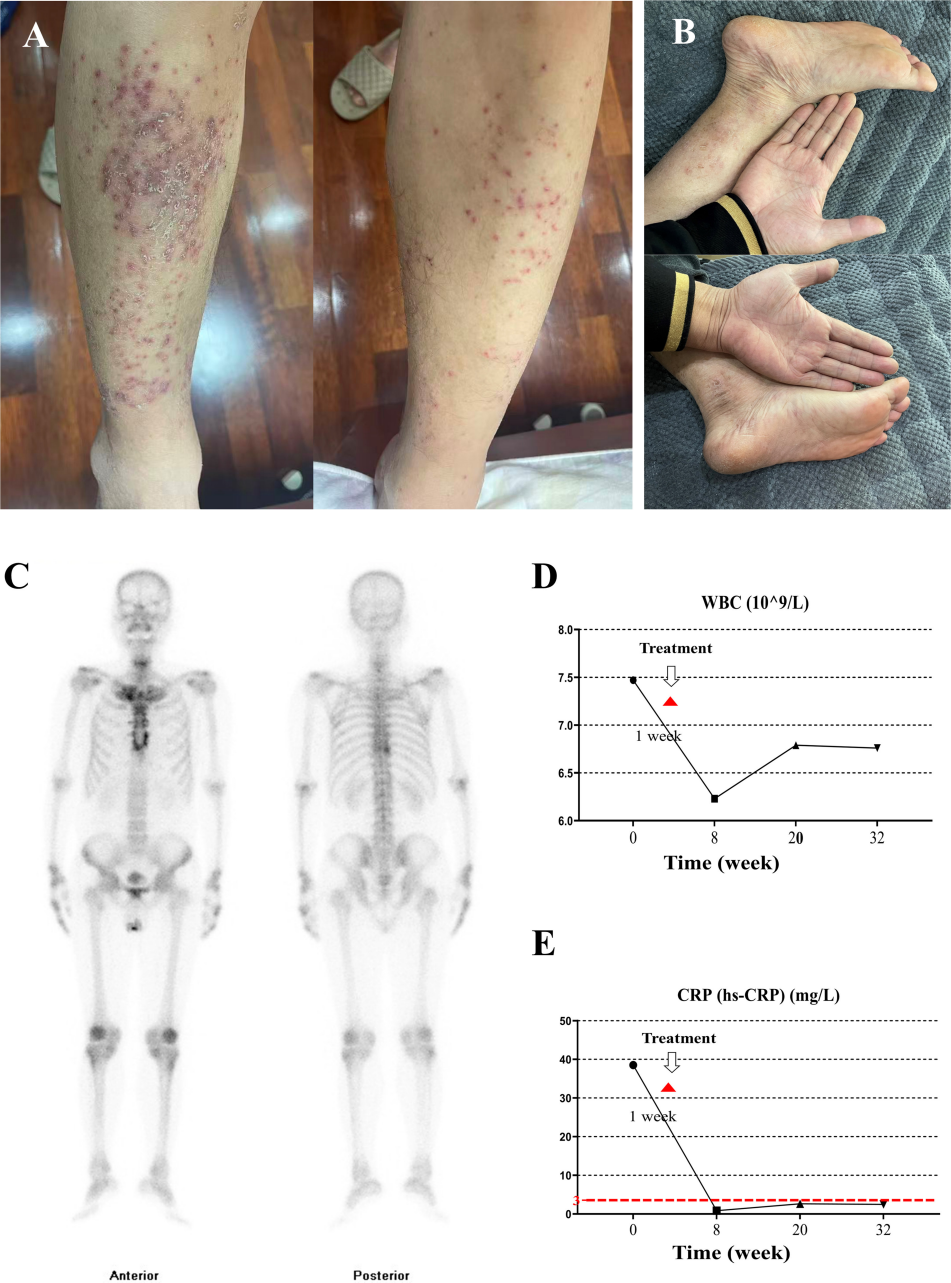
Clinical manifestations, bone scintigraphy, and laboratory findings. **(A)** Pustular lesions on both lower extremities with partial crusting. **(B)** Skin lesions after treatment, showing marked improvement and minimal residual crusting. **(C)** Bone scintigraphy shows increased tracer uptake in the manubrium, sternal body, bilateral first sternocostal joints and sternoclavicular joints, forming the characteristic “bull’s head sign” (arrows). **(D)** Serial white blood cell counts during follow-up. **(E)** Changes in high-sensitivity C-reactive protein levels after treatment; the red dashed line indicates the upper limit of normal.

In October 2022, the patient underwent a routine physical examination at our hospital, where imaging and laboratory tests were performed. Chest computed tomography (CT) (tube voltage 120 kV, tube current 120 mAs, slice thickness 0.65 mm, DLP 339–341 mGy⋅cm) showed multiple moth-eaten osteolytic lesions with reactive sclerosis and hyperostosis involving the sternum, medial ends of both clavicles, anterior portions of the first and second ribs bilaterally, and the T6–T10 and T12–L2 vertebral bodies (Figures 1A–E). Laboratory testing revealed elevated inflammatory markers, including high-sensitivity C-reactive protein, as well as abnormal rheumatological and immunological results (Figure 2E). Serum amyloid A (SAA) was elevated (79.40 mg/L), immunoglobulin A (IgA) was elevated (4.68 g/L), complement 3 (C3) was elevated (1.31 g/L), and complement 4 (C4) was elevated (0.40 g/L). In contrast, levels of anti-streptolysin O (ASO), anti-deoxyribonuclease B (anti-DNase B), rheumatoid factor (RF), and immunoglobulin M (IgM) were normal. Liver function tests, urea/creatinine levels, and tumor marker (CEA, AFP, SCC, CA199, CA125) were also within normal limits. Because an inflammatory rheumatic disorder was suspected, the patient was referred for further assessment.

The patient was admitted to the Department of Rheumatology and Immunology in November 2022. Physical examination showed tenderness over the sternoclavicular joints, with no limitation of spinal movement. Pustular lesions were present on both hands and lower legs, particularly on the calves (Figure 2A). Considering the sternoclavicular joint tenderness, pustular skin lesions on the extremities, imaging evidence of reactive sclerosis and hyperostosis involving the sternum, clavicles, ribs, and vertebrae, elevated inflammatory markers, and the previously reported absence of tuberculosis, infection, and malignancy on external bone biopsy, we suspected SAPHO syndrome. Initial treatment was started with methotrexate 10 mg once weekly and celecoxib 200 mg once daily. Meanwhile, whole-body bone scintigraphy and a comprehensive rheumatological autoantibody panel were arranged to help exclude alternative diagnoses, including anti-nuclear antibody (ANA), anti-double-stranded DNA antibody (Anti-dsDNA), anti-histone antibody (AHA), anti-nucleosome antibody (AnuA), C1q antibody (C1q Ab), anti-Sjögren’s syndrome A antibody (Anti-SSA), anti-Sjögren’s syndrome B antibody (Anti-SSB), anti-Smith antibody (Anti-Sm), anti-Jo-1 antibody (Anti-Jo-1), anti-ribonucleoprotein antibody (Anti-RNP), anti-Scleroderma 70 antibody (Anti-Scl-70), and anti-centromere antibody (ACA).

To confirm the extent of skeletal involvement, whole-body bone scintigraphy was performed using a 740 MBq (20 mCi) intravenous injection of 99mTc-methylene diphosphonate (MDP). Whole-body images were acquired 3 h post-injection. The scan showed increased tracer uptake in the manubrium, sternal body, bilateral first sternocostal joints and sternoclavicular joints, forming the “bull’s head sign” (Figure 2C). There was no scintigraphic evidence of sacroiliac joint involvement or spondylitis. Notably, the patient lacked key features such as inflammatory back pain with morning stiffness, dactylitis, uveitis, and objective evidence of sacroiliitis, demonstrating a lack of compliance with the SPARTAN 2025 criteria (9), which allowed us to further exclude SpA. Based on the overall clinical picture, imaging findings, and exclusion of infection, malignancy, and other spondyloarthritides, SAPHO syndrome was diagnosed according to the Kahn criteria (10, 11). After the diagnosis was established, adalimumab 40 mg every 2 weeks was added, methotrexate and celecoxib were continued.

The patient was followed in our rheumatology and immunology department at 3, 5, and 8 months after treatment initiation, with no loss to follow-up during this period. He adhered well to treatment and attended regular rheumatology visits as scheduled. Inflammatory markers remained within normal limits at each follow-up, with no significant fluctuation (Figures 2D,E). Thereafter, the patient returned to a local hospital for regular review. At 12 months after his initial visit to our hospital, the health management center contacted him by telephone for follow-up, and he reported satisfactory recovery, with marked improvement in chest and osteoarticular pain and near-complete resolution of the skin lesions (Figure 2B). No treatment-related adverse effects or disease recurrence were reported during the follow-up period. Follow-up imaging was limited to routine CT, which showed stable bone lesions without progression. Repeat bone scintigraphy was not performed because the patient had achieved sustained clinical improvement and normalization of inflammatory markers, and additional nuclear imaging was not considered necessary for management.

## Literature search

To better understand the clinical trajectory of delayed diagnosis in SAPHO syndrome and to place our case in context (summarized in Table 2), we performed a targeted literature search. PubMed/MEDLINE was searched from inception to December 2025 using the following strategy: (“SAPHO syndrome” OR “synovitis acne pustulosis hyperostosis osteitis”) AND (“misdiagnosis” OR “delayed diagnosis” OR “diagnostic error”). In total, 35 articles were identified. Titles and abstracts were screened, and full texts were reviewed when relevant. We included adult case reports or case series (≥18 years) with a definitive diagnosis of SAPHO syndrome and an explicitly described initial misdiagnosis. Articles were excluded if they were not published in English, lacked sufficient detail regarding the diagnostic timeline, or focused exclusively on pediatric chronic non-bacterial osteomyelitis. Thirteen articles were retained for final analysis.

**TABLE 2:** Clinical features and diagnostic methods of SAPHO syndrome patients misdiagnosed with other diseases in the literature.

Patient and ref	Sex	Age	Symptoms		examination	Initial Diagnosis	Final Diagnosis	Time to diagnosis
			Skin	Bone				
1. This case	Male	51	Pustules on both hands and feet.	Chest pain and low back pain.	1. Inflammatory markers are significantly elevated. 2. CT scan showed multiple worm-eaten bony destructions in the sternum, thoracic vertebrae and bilateral clavicles.	1. Tuberculosis. 2. Pyogenic spondylitis.	SAPHO syndrome	5 years
2. Ghita et al. (20)	Male	40	Facial and Palmo-plantar pustulosis.	3 months history of cervical inflammatory pain and recurrent torticollis.	CRP = 56 mg/L, ESR = 120 mm/h (Inflammatory markers are significantly elevated).	Staphylococcal infection	SAPHO syndrome with C1–C2 spondylodiscitis.	4 months
3. Nakamura et al. (21)	Female	60	No significant skin lesions.	Severe low-back and leg pain after treatment for tuberculosis.	1. Tomography/computed tomography showed uptake in the cervical pines, lumbar spines, and sacroiliac joints. 2. Pathological findings showed nonspecific inflammation and fibrous hypertrophy of the bone marrow.	Tuberculosis	SAPHO syndrome	/
4. Michaele et al. (14)	Female	56	Multiple nodular and ulcerative cutaneous lesions in the arms and chest wall.	Stiffness and pain in right shoulder.	1. Skeletal hy perostosis, particularly of the anterior chest wall, with deformity and hyperostosis of the sternocostal and sternoclavicular joints. 2. Improved with the institution of antimicrobial therapy.	Seronegative rheumatoid arthritis.	SAPHO syndrome	20 years
5. Saira et al. (15)	Male	40	No significant skin lesions.	Right foot pain on weight bearing.	Biopsy shows chronic inflammation without evidence of infection or malignancy.	Bacterial Osteomyelitis	SAPHO syndrome	4 months
6. Charles et al. (24)	Male	51	No significant skin lesions.	Severe inflammatory pain in the thoracic spine.	Mild inflammation (CRP = 15 mg/L, ESR = 26 mm/h).	1. Infectious Spondylitis. 2. Malignant Tumors. 3. Paget’s Disease. 4. Seronegative. 5. Spondyloarthritis.	SAPHO syndrome	10 months
7. Bijit et al. (25)	Female	42	Severe acne with pus formation and hidradenitis suppurativa.	Sudden onset of pain in the waist and lower back.	Bone scintigraphy showed “bull’s head.”	Other diseases	SAPHO syndrome	3 years
8. Zeng et al. (35)	Male	22	Severe facial acne extending to the trunk.	Left shoulder pain and a lowgrade fever.	Bone scintigraphy showed “bull’s head.”	Acute exacerbation of chronic osteomyelitis of the left clavicle.	SAPHO syndrome	/
9. Fernando et al. (37)	Female	66	No significant skin lesions.	Back pain for 4 months.	CT scan showed marked sclerosis and subcortical bone irregularity.	Spinal bone metastasis	SAPHO syndrome	4 months
10. Bijit et al. (25)	Male	42	No significant skin lesions.	Having pain in the upper back.	Bone scintigraphy showed “bull’s head.”	Malignancy in a case of ankylosing spondylitis.	SAPHO syndrome	5 years
11. Fernando et al. (37)	Female	56	No significant skin lesions but palmar and plantar pustulosis two years.	A 6-month history of back pain.	Bone scintigraphy showed “bull’s head.”	Bone metastases	SAPHO syndrome	2 years
12. Awadh et al. (26)	Male	37	Pustular skin eruptions.	Back and neck pain.	Bone scintigraphy showed “bull’s head.”	Ankylosing Spondylitis	SAPHO syndrome	7 years
13. Wang et al. (18)	Female	59	Pustular changes on the plantar surface of the left foot.	Low back pain and recurrent fever.	1. ESR 81 mm/h, CRP 15.58 mg/L. 2. ECT showed increased radionuclide uptake in bilateral first sternocostal joints (“bull’s head sign”). 3. Biopsy showed no pathogen.	Chronic spinal infection or pyogenic spondylitis	SAPHO syndrome	2 months
14. Karadag-Saygi et al. (38)	Female	53	Bordered erythe-matous desquamated plaques psoriatic-like skin lesions.	Pain and stiffness of the neck and shoulders and low back pain.	1. Bone scan showing increased uptake in the sternocostoclavicular regions. 2. Dense fibrosis and chronic inflammation in adipose tissue.	Malignancy	SAPHO syndrome	4 years
15. Yang et al. (19)	Female	68	Palmoplantar pustulosis.	Cervical and lumbosacral region pain.	1. Inflammatory markers were elevated. 2. MRI showed bone marrow edema in umber and cervical spine. 3. Biopsy exclusion of infection and malignancy.	Spinal infection	SAPHO syndrome	2 months
16. Yang et al. (19)	Female	39	Palmoplantar pustulosis and psoriasis.	Cervical region pain.	1. ET/CT revealed osteolytic bone destruction with sclerosis. 2. Biopsy exclusion of infection and malignancy.	Cervical spinal infection	SAPHO syndrome	2 months
17. Yang et al. (19)	Female	62	Palmoplantar pustulosis.	Cervical and thoracic region pain, peripheral joint pain.	1. CRP 45.78 mg/L (elevated), interleukin-6 (IL-6) 10.3 pg/mL (elevated), ESR 53 mm/h (elevated), and IgA 4.79 g/L (elevated). 2. Biopsy exclusion of infection and malignancy. 3. ET/CT imaging revealed multiple osteolytic bone destruction with sclerosis in the costovertebral joint.	Spinal infection	SAPHO syndrome	6 years
18. Kubo et al. (39)	Female	69	Rash on both palms.	Severe back pain.	Bone scan showed abnormal uptake in multiple vertebrae and left sternoclavicular joint.	Bone metastases	SAPHO syndrome	4 months

## Discussion

SAPHO syndrome remains a diagnostic challenge because of its rarity, heterogeneous presentation, and lack of specific biomarkers. Diagnostic delay of several years is common (7, 12), and patients are frequently misclassified as having infection, inflammatory arthritis, or malignancy, leading to prolonged pain, repeated investigations, and unnecessary treatment (13–15). When unusual skin lesions such as palmoplantar pustulosis, hidradenitis suppurativa, or severe acne are present (16, 17), misdiagnosis as psoriasis or other dermatological conditions is also common (17). In our patient, the diagnosis was delayed for 5 years, during which he underwent multiple consultations, invasive procedures, and prolonged empiric anti-infective therapy without benefit.

Misdiagnosis most commonly occurs when SAPHO syndrome mimics infection, especially spinal tuberculosis, infectious spondylitis (18, 19). Vertebral destruction, pain, and elevated inflammatory markers may prompt prolonged anti-tuberculosis treatment or even surgery before sterile osteitis is recognized (15, 20, 21). The confusion is especially likely when spinal lesions are present without obvious anterior chest wall involvement or skin disease (22, 23). In contrast, sclerosis and hyperostosis of the sternoclavicular or sternocostal region should raise suspicion for SAPHO syndrome (22). However, because tuberculosis is common in many regions, SAPHO syndrome is not always considered early in the differential diagnosis. In the present case, delayed diagnosis was partly attributable to limited local medical resources and lack of disease awareness.

SAPHO syndrome may also be confused with other rheumatologic disorders, particularly spondyloarthritis (SpA) and rheumatoid arthritis (RA). Patients with bone and joint pain and elevated inflammatory markers are often labeled as having seronegative RA or ankylosing spondylitis (14, 24–26). Distinguishing features of SAPHO syndrome include frequent involvement of the anterior chest wall, particularly the sternoclavicular joints. Although typical SpA is often associated with bilateral sacroiliitis and “bamboo spine” (27), these are not its only features. Careful differentiation from SAPHO syndrome is therefore needed. SpA covers a broad clinical spectrum, including inflammatory back pain, peripheral arthritis, enthesitis, dactylitis, and specific extra-articular manifestations (9). HLA-B27 positivity is inconsistent in SAPHO syndrome and is reported only in a small proportion of patients, whereas it is common in classic SpA (7, 28). HLA-B27 positivity has been documented in only a small proportion of patients, with reported rates ranging from 3 to 5% (12, 27). By comparison, the HLA-B27 positivity rate in classic SpA can be as high as 90% (29). RA is usually characterized by synovitis of the small joints and seropositivity for RF or anti-CCP antibodies, negative RF and anti-CCP results argue against RA (30).

When isolated bone lesions predominate and skin manifestations are subtle or absent, SAPHO syndrome may be mistaken for bone tumor, malignancy, or non-specific osteomyelitis (31). Clinically, SAPHO syndrome can be divided into forms with skin manifestations and forms presenting mainly as sterile osteitis, often within the spectrum of chronic non-bacterial osteomyelitis (32–34). Young men presenting with isolated bone pain seem particularly vulnerable to this diagnostic confusion (35). CT may show osteolytic lesions, sclerosis, or hyperostosis that are initially interpreted as malignant or infectious bone disease (24, 25, 36–39).

Imaging plays an important supportive role in SAPHO syndrome, especially when skin findings are absent or delayed. Bone scintigraphy is more sensitive than radiography or CT for detecting multifocal or subclinical bone involvement (40–42). The “bull’s head sign,” caused by increased tracer uptake in the sternoclavicular region, is a useful clue in the proper clinical context (40, 41). However, it is not specific to SAPHO syndrome and may also be seen in other conditions, including malignancies and ankylosing spondylitis (43, 44). Moreover, the sign is absent in many confirmed cases. One study found it in only 22.9% of 48 patients with SAPHO syndrome (45). Therefore, the bull’s head sign should be interpreted as a supportive, not definitive, finding and must be integrated with the clinical presentation and exclusionary evaluation.

SAPHO syndrome is often misdiagnosed because of its low prevalence, heterogeneous clinical presentation, and lack of specific diagnostic markers. In our case, the diagnosis was supported by osteitis, pustular skin lesions, suggestive imaging findings, and biopsy results excluding infection and malignancy. Notably, to our knowledge, this may be the first case in which SAPHO syndrome was suspected through a routine health check-up and ultimately confirmed after further comprehensive evaluation. The routine examination played an important role in prompting reevaluation after years of delayed diagnosis. Treatment with adalimumab combined with methotrexate and NSAIDs led to marked clinical and laboratory improvement, showing that early diagnosis can improve outcomes and help avoid unnecessary invasive procedures, antibiotics, and antituberculosis therapy.

This case and our review indicate that SAPHO syndrome may be considered when chronic sterile osteitis coexists with pustular skin lesions. Diagnosis should rely on a combination of clinical findings rather than any single criterion alone. Future efforts should focus on improving clinician awareness, especially for atypical presentations, and on promoting earlier comprehensive evaluation using integrated clinical, imaging, and exclusionary evidence. Improved awareness and timely evaluation are essential to reduce diagnostic delay, avoid inappropriate treatment, and improve patient outcomes.

## Data Availability

The raw data supporting the conclusions of this article will be made available by the authors, without undue reservation.

## References

[1] Chamot AM Benhamou CL Kahn MF Beraneck L Kaplan G Prost A. [Acne-pustulosis-hyperostosis-osteitis syndrome. Results of a national survey. 85 cases]. *Rev Rhum Mal Osteoartic.* (1987) 54:187–96. 2954204

[2] Iva R. SAPHO syndrome: a review. *J Child Orthop.* (2015) 9:19–27. 10.1007/s11832-014-0627-7 25585872 PMC4340847

[3] Marcello G Matteo C Alfonso M Francesco T. SAPHO syndrome and infections. *Autoimmun Rev.* (2008) 8:256–9. 10.1016/j.autrev.2008.07.030 18721907

[4] Alexander PR. SAPHO syndrome: is a range of pathogen-associated rheumatic diseases extended? *Arthritis Res Ther.* (2009) 11:131. 10.1186/ar2837 19895718 PMC3003498

[5] Chen L Hesong W Haixu J Yuming S Guangrui H Kai Y et al. Family aggregation and prevalence of other autoimmune diseases in SAPHO syndrome. *Heliyon.* (2023) 9:e21541. 10.1016/j.heliyon.2023.e21541 38027688 PMC10654150

[6] Mario F Juela L Riccardo DL Caterina M Marco G Serena G et al. What is new and what is next for SAPHO syndrome management: a narrative review. *J Clin Med.* (2025) 14:1366. 10.3390/jcm14041366 40004896 PMC11856149

[7] Hayem G Bouchaud-Chabot A Benali K Roux S Palazzo E Silbermann-Hoffman O et al. Sapho syndrome: a long-term follow-up study of 120 cases. *Semin Arthritis Rheum.* (2000) 29:159–71. 10.1016/s0049-0172(99)80027-4 10622680

[8] Petra Z Nigel C. Synovitis, acne, pustulosis, hyperostosis, and osteitis (SAPHO) syndrome - a challenging diagnosis not to be missed. *J Infect.* (2016) 72:S106–14. 10.1016/j.jinf.2016.04.030 27263075

[9] Victoria N Alexandre S Dafne C Xenofon B. Axial spondyloarthritis. *Lancet.* (2025) 405:159–72. 10.1016/S0140-6736(24)02263-3 39798984

[10] Kahn MF. Proposed classification criteria of SAPHO syndrome. *American College of Rheumatology 67th Annual Scientific Meeting.* (2003).

[11] Kahn MF Khan MA. The SAPHO syndrome. *Baillieres Clin Rheumatol.* (1994) 8:333–62. 10.1016/s0950-3579(94)80022-7 8076391

[12] Yihan C Chen L Wenrui X Xia W Xiaochuan S Weihong Z et al. Spinal and sacroiliac involvement in SAPHO syndrome: a single center study of a cohort of 354 patients. *Semin Arthritis Rheum.* (2019) 48:990–6. 10.1016/j.semarthrit.2018.09.004 30935678

[13] Sophie WSL Eve R Christian H. Treatment and monitoring of SAPHO syndrome: a systematic review. *RMD Open.* (2023) 9:e003688. 10.1136/rmdopen-2023-003688 38151265 PMC10753757

[14] Michaele Francesco C Nisha B Hannah K Anthony S Lorna A Carlos F. Clinical spectrum of *Cutibacterium acnes* infections: the SAPHO syndrome. *IDCases.* (2023) 32:e01784. 10.1016/j.idcr.2023.e01784 37214184 PMC10195882

[15] Saira EAK Umaima MK Maira DN. A case report on the atypical symptoms of the synovitis, acne, pustulosis, hyperostosis, and osteitis (SAPHO) syndrome: could covid-19 be a cause? *Cureus.* (2023) 15:e41498. 10.7759/cureus.41498 37551213 PMC10404349

[16] Francesco C Angelo Z Elisa G Gianfranco F. Clinical heterogeneity of SAPHO syndrome: challenging diagnose and treatment. *Clin Rheumatol.* (2017) 36:2151–8. 10.1007/s10067-017-3751-1 28725947

[17] Chen W Ito T Lin SH Song Z Al-Khuzaei S Jurik AG et al. Does SAPHO syndrome exist in dermatology? *J Eur Acad Dermatol Venereol.* (2022) 36:1501–6. 10.1111/jdv.18172 35462435

[18] Shuai W Shiyong W Zhengqi C. Misdiagnosed with spinal infection instead of SAPHO syndrome: a case report and literature review. *Front Immunol.* (2025) 16:1683214. 10.3389/fimmu.2025.1683214 41181134 PMC12571820

[19] Xiaoyan Y Jina G Chengjun Z Danmei P Hongchao C Qinbin Q et al. SAPHO syndrome misdiagnosed as spinal infection: a case series. *Int Med Case Rep J.* (2025) 18:1329–41. 10.2147/IMCRJ.S526910 41127865 PMC12537524

[20] Ghita H Ahlam B Imane EB Meryem OI Najat CI Ahmed O et al. C_1_-C_2_ spondylodiscitis in an adult with SAPHO syndrome: an unusual presentation. *Rheumatol Int.* (2009) 32:445–7. 10.1007/s00296-009-1274-z 20024557

[21] Jun-ichiro N Katsutaka Y Naoto M Tomoyuki S. A case of SAPHO syndrome with destructive spondylodiscitis suspicious of tuberculous spondylitis. *Mod Rheumatol.* (2009) 20:93–7. 10.1007/s10165-009-0234-5 19830381

[22] Hanna P Marek B. Sapho syndrome: pathogenesis, clinical presentation, imaging, comorbidities and treatment: a review. *Postepy Dermatol Alergol.* (2022) 38:937–42. 10.5114/ada.2020.97394 35125997 PMC8802951

[23] Ravindra Kumar G Dilip Singh S. Spinal tuberculosis: a review. *J Spinal Cord Med.* (2011) 34:440–54. 10.1179/2045772311Y.0000000023 22118251 PMC3184481

[24] Charles C Céline C Véronique M Didier C Anne M Jacques Yves N. Isolated thoracic spine lesion: is this the presentation of a SAPHO syndrome? A case report. *Eur Spine J.* (2004) 14:711–5. 10.1007/s00586-004-0791-4 15480825 PMC3489227

[25] Bijit Kumar K Ananta Kumar N Shrinath B Dinesh S. Diagnosing the SAPHO syndrome: a report of three cases and review of literature. *Clin Rheumatol.* (2013) 32:1237–43. 10.1007/s10067-013-2251-1 23604547

[26] Nabaa Ihsan A Faiq IG Ahmed Dheyaa A Hashim Talib H Mustafa Najah A Reem Abbas H. Unusual cause of inflammatory backache: SAPHO syndrome. *Int J Rheum Dis.* (2023) 27:e14878. 10.1111/1756-185X.14878 37592395

[27] Victoria F Mitsumasa K Tetsuya T Ori E Philip SH. Pro and contra: is synovitis, acne, pustulosis, hyperostosis, and osteitis (SAPHO) a spondyloarthritis variant? *Curr Opin Rheumatol.* (2022) 34:209–17. 10.1097/BOR.0000000000000884 35699334

[28] Faisal A Anne T Zuzana T Marion C Sylvain M Sandrine M et al. The SAPHO syndrome: a single-center study of 41 adult patients. *J Rheumatol.* (2014) 42:329–34. 10.3899/jrheum.140342 25512472

[29] Nguyen MT Borchers A Selmi C Naguwa SM Cheema G Gershwin ME. The SAPHO syndrome. *Semin Arthritis Rheum.* (2012) 42:254–65. 10.1016/j.semarthrit.2012.05.006 23153960

[30] Céline G Margarita H Pascale N Rodrigo F Caroline B Emilie Q et al. Prevalence of autoantibodies in SAPHO syndrome: a single-center study of 90 patients. *J Rheumatol.* (2010) 37:639–43. 10.3899/jrheum.090863 20110527

[31] Cunhao S Fanzhang M Aijie Y Zhimin L Fan B Litao Z et al. A case of SAPHO syndrome with wrist involvement as the first presentation and response to tofacitinib therapy. *Joint Bone Spine.* (2025) 92:105960. 10.1016/j.jbspin.2025.105960 40935121

[32] Schilling F Kessler S. [SAPHO syndrome: clinico-rheumatologic and radiologic differentiation and classification of a patient sample of 86 cases]. *Z Rheumatol.* (2000) 59:1–28. 10.1007/s003930050001 10769419

[33] Christian MH Henner M Christiane R Hermann JG. New insights into adult and paediatric chronic non-bacterial osteomyelitis CNO. *Curr Rheumatol Rep.* (2020) 22:52. 10.1007/s11926-020-00928-1 32705386 PMC7378119

[34] Dan Yongdong Z Liza M Gabriele H Christian MH. Chronic nonbacterial osteomyelitis (CNO) and chronic recurrent multifocal osteomyelitis (CRMO). *J Transl Autoimmun.* (2021) 4:100095. 10.1016/j.jtauto.2021.100095 33870159 PMC8040271

[35] Wanghui Z Yuqiao Z Xiaobin Z Ruijun F. Misdiagnosis of SAPHO syndrome as chronic infectious myelitis: a rare case report. *Asian J Surg.* (2025) 48:1830–2. 10.1016/j.asjsur.2024.09.083 39332955

[36] Zheng W Zhang J Zhang R. Postpartum lumbopelvic pain could be SAPHO syndrome: a case report. *Front Immunol.* (2025) 16:1614945. 10.3389/fimmu.2025.1614945 40881705 PMC12380585

[37] Fernando Luiz RD François D Rômulo TNDG Bárbara CM Victor KTDF. Synovitis, acne, pustulosis, hyperostosis, osteitis (SAPHO) syndrome mimicking bone metastases in the spine: a presentation of two cases and literature review. *Cureus.* (2024) 16:e64974. 10.7759/cureus.64974 39161490 PMC11333024

[38] Evrim KS Hakan Gunduz O Gulistan G Gulseren A. Sapho syndrome: misdiagnosed and operated. *Acta Reumatol Port.* (2008) 33:460–3. 19078862

[39] Yuko K Kimiteru I Yutaka F Tatsuya Y Masahiko K. Case report: SAPHO syndrome mimicking bone metastases during treatment with pembrolizumab for non-small cell lung cancer. *Front Med (Lausanne).* (2021) 8:679111. 10.3389/fmed.2021.679111 34368186 PMC8339402

[40] Steven S Hartley MS Jonathan K. Imaging for synovitis, acne, pustulosis, hyperostosis, and osteitis (SAPHO) syndrome. *Rheum Dis Clin North Am.* (2016) 42:695–710. 10.1016/j.rdc.2016.07.011 27742022

[41] Hidetomo H Seiji K Shuji N Akiko S Fumihiro T Akihiro M et al. Imaging features in patients with SAPHO/CRMO: a pictorial review. *Jpn J Radiol.* (2020) 38:622–9. 10.1007/s11604-020-00953-1 32356235

[42] Anne Grethe J Rikke Fuglsang K Paolo S Philip R James T. SAPHO and CRMO: the value of imaging. *Semin Musculoskelet Radiol.* (2018) 22:207–24. 10.1055/s-0038-1639469 29672809

[43] Jianming N Ping T. An unusual bone metastasis mimicking SAPHO (synovitis, acne, pustulosis, hyperostosis, and osteitis) syndrome on bone scintigraphy. *Clin Nucl Med.* (2015) 41:173–5. 10.1097/RLU.0000000000001061 26505860

[44] Guglielmi G Cascavilla A Scalzo G Salaffi F Grassi W. Imaging of sternocostoclavicular joint in spondyloarthropaties and other rheumatic conditions. *Clin Exp Rheumatol.* (2009) 27:402–8. 19604431

[45] Zhanli F Meng L Ziao L Yan F Jianhua Z Xuchu Z et al. Is the bullhead sign on bone scintigraphy really common in the patient with SAPHO syndrome? A single-center study of a 16-year experience. *Nucl Med Commun.* (2015) 37:387–92. 10.1097/MNM.0000000000000451 26619395

